# Measuring Neighborhood Socioeconomic Status from Sky and Street: An Ensemble Learning Framework

**DOI:** 10.1177/27541231261473758

**Published:** 2026-08-20

**Authors:** Yan Li, Ying Long, Esra Suel, Yuyang Zhang, Brian E. Robinson, Alicia Catherine Cavanaugh, Nanxi Su, Majid Ezzati

**Affiliations:** a School of Architecture, Tsinghua University, Beijing, China; b School of Architecture and Hang Lung Center for Real Estate, Key Laboratory of Ecological Planning & Green Building, Ministry of Education, Tsinghua University, Beijing, China; c Centre for Advanced Spatial Analysis, University College London, London, UK; d Department of Geography, McGill University, Montreal, Canada; e MRC Centre for Environment and Health, School of Public Health, Imperial College London, London, UK

**Keywords:** neighborhood socioeconomic status (nSES), geospatial artificial intelligence (geoAI), satellite image, street view image, ensemble learning, multi-view fusion, model interpretability, neighborhood social inequality

## Abstract

Fine-scale, geocoded neighborhood socioeconomic status (nSES) data are foundational inputs for urban studies and spatial inequality research, yet remain scarce in emerging economies that together account for over 70% of the global population. Here, we use publicly available satellite and street view imagery to estimate nSES at fine spatial resolution, proposing an ensemble regression framework that unites multi-view images to measure intra-urban inequality. We estimate eight indicators — spanning household registration (hukou), housing, income, employment, and education — as numeric values rather than broad quantile bands, enabling direct cross-neighborhood comparison. Family and individual SES from a representative survey serve as training data. The proposed fusion model outperforms single ensemble regression models and single-view image-based models in mean absolute percentage error. To interpret image-based prediction, we apply SHAP-based feature attribution and modality-contribution analysis, identifying the built-environment features that drive predictions and revealing that satellite and street view imagery play complementary rather than redundant roles. Neighborhood inequalities at traffic analysis zone and township scales in central Beijing are visualized for the first time. We further transfer the trained model to Shanghai, where no comparable public nSES dataset exists, providing a limited proof-of-concept against real-estate data for two of the eight indicators and outlining a replicable pathway for data-scarce cities.

## 1 Introduction

Neighborhood socioeconomic status (nSES) captures the aggregate economic, educational, and housing conditions of a residential area and is a core construct in urban studies, regional science, and spatial inequality research (Sampson, Morenoff, & Gannon-Rowley, 2002). Accurate, fine-scale nSES measurements underpin a wide range of urban analyses, from residential segregation and housing affordability to the spatial targeting of public services and the design of community-level interventions. However, in most emerging economies — which together account for over 70% of the global population — such fine-scale data are either unavailable or restricted to coarse administrative aggregates, severely limiting intra-urban analysis and cross-city comparison.

The scarcity of fine-scale nSES data has both institutional and technical roots. Existing nSES datasets, typically derived from national censuses or dedicated surveys such as the American Community Survey, are updated only every five to ten years and require substantial resources for data collection (Yang et al., 2014). In China and many other emerging economies, official statistics are released only at the city or township level, and no data are available at finer scales. Even where finer data exist, privacy restrictions often prevent their public release in raw form; for example, the Surveillance, Epidemiology, and End Results (SEER) database reports nSES indicators only as quantile bands rather than true values, making them unintuitive for cross-regional comparison and unsuitable for many downstream applications. Together, these constraints leave a substantial gap between the spatial resolution at which urban processes operate (the neighborhood) and the resolution at which nSES data are actually available (the township or city).

Advances in geospatial artificial intelligence (geoAI) have opened a promising alternative: inferring nSES directly from publicly available imagery. A growing body of work has shown that machine learning models can extract socioeconomically relevant signals from a variety of urban data sources, including mobile signaling, social media, and e-commerce data for predicting regional population and consumption (Dong, Ratti, & Zheng, 2019; Long & Thill, 2015); daytime and nighttime satellite imagery for capturing intra- and inter-urban economic dynamics (Wang, Sutton, & Qi, 2019); and street view imagery for inferring income, education, and housing conditions at the neighborhood level (Fan, Zhang, Loo, & Ratti, 2023; Gebru et al., 2017; Naik, Kominers, Raskar, Glaeser, & Hidalgo, 2017; Suel, Polak, Bennett, & Ezzati, 2019). Beyond socioeconomic inference, street view imagery has also been used to measure other neighborhood-level attributes such as physical disorder (Chen, Chen, Li, Zhang, & Long, 2023), storefront vacancy (Li & Long, 2024), and abandoned buildings in shrinking cities (Li, Meng, Zhao, Li, & Long, 2023), further demonstrating the capacity of ground-level imagery to capture fine-scale built-environment conditions. Among these data sources, satellite and street view imagery are particularly attractive for emerging-economy contexts because they offer wide spatial coverage, low acquisition cost, and — crucially — independence from the digital infrastructure that social-sensing data sources require. Recent studies have further leveraged deep learning and spatio-temporal large language models to interpret urban environments at unprecedented scale (Li et al., 2024; Feng et al., 2025). Notably, time-series street view imagery has been used to track the temporal evolution of urban physical conditions at a national scale (Ma, Li, & Long, 2025), indicating that the visual signals captured by such imagery are not static snapshots but can reflect longitudinal urban change. Table 1 summarizes the principal data sources used in prior nSES studies, the nSES dimensions they have been applied to, and the representative works in each category.

As Table 1 makes clear, despite the diversity of data sources explored in prior work, two recurring limitations constrain the practical value of existing image-based nSES studies. First, most prior work predicts only coarse quantile bands (e.g., income deciles) of a single indicator rather than continuous numeric values across multiple dimensions, which limits direct cross-neighborhood comparison and obscures finer gradients of inequality. To our knowledge, only one prior study has attempted to combine satellite and street view imagery for nSES prediction (Suel, Bhatt, Brauer, Flaxman, & Ezzati, 2021), and even there the targets remain quantile bands rather than continuous values. Second, existing studies treat image-to-nSES mapping as a black-box regression problem, reporting accuracy metrics without explaining which visual features of the built environment drive the predictions or how satellite and street view imagery contribute differently across nSES dimensions. For an urban-science audience, this lack of interpretability is a serious limitation: knowing that a model predicts income accurately is far less useful than knowing which built-environment features (façade condition, building density, greenery, sky openness) it relies on, and whether the satellite and street view perspectives are genuinely complementary or merely redundant.

To address both gaps, this study develops a multi-view ensemble learning framework that (i) predicts continuous numeric values for eight nSES indicators across five dimensions and (ii) explicitly exposes the contribution of individual visual features and image modalities through SHAP-based interpretation. The eight indicators are: percentage of households with a Beijing hukou (institutional status); percentage of the tertiary-educated population and mean years of schooling (human capital); percentage of the high-paying working population (labor market); mean household income (economic resources); and mean floor area, mean floor area per capita, and percentage of commercial housing (physical housing conditions). Throughout this paper, multi-view fusion refers to the combined use of satellite (overhead) and street view (ground-level) imagery within a single predictive model, such that features from both perspectives jointly inform the nSES estimate for each neighborhood. The 2015 Beijing Household Travel Survey, a representative survey covering 418 of the 941 traffic analysis zones (TAZs) in central Beijing, is used as ground truth for training and validation.

The main contributions of this study are:

(1) A method for predicting continuous, multi-dimensional nSES from imagery. Existing image-based nSES studies predominantly predict coarse quantile bands of a single indicator. We show that a stacked multi-view ensemble can recover numeric values across eight indicators spanning five distinct nSES dimensions, enabling finer-grained and more interpretable cross-neighborhood comparisons than quantile-based approaches.
(2) Interpretability of the image–nSES relationship. Prior work has largely treated image-based nSES prediction as a black-box regression task. By coupling the ensemble with SHAP-based feature attribution and modality-contribution analysis, we quantify which visual features (e.g., building density, greenery ratio, façade heterogeneity) drive predictions for each nSES dimension, and we show that satellite and street view imagery play complementary rather than redundant roles across different indicators.
(3) A reproducible pipeline for cities lacking fine-scale survey data. We deliver a fine-scale, full-coverage nSES map for the central urban area of Beijing — including the 523 TAZs without survey coverage — and demonstrate cross-city transferability to Shanghai, where no comparable public nSES dataset exists. This establishes a replicable template for constructing fine-scale nSES datasets in cities where traditional census data are unavailable or outdated.

The remainder of this paper is organized as follows. Section 2 describes the study area, survey data, nSES indicator definitions, and image data sources. Section 3 presents the proposed multi-view ensemble framework, organized around three design questions concerning feature extraction, ensembling, and modality fusion. Section 4 reports prediction accuracy, the new interpretability analysis, and the Shanghai transfer experiment. Section 5 discusses the implications and limitations of the work, and Section 6 concludes.

## 2 Data and materials

### 2.1 Study area

**Figure 1:**
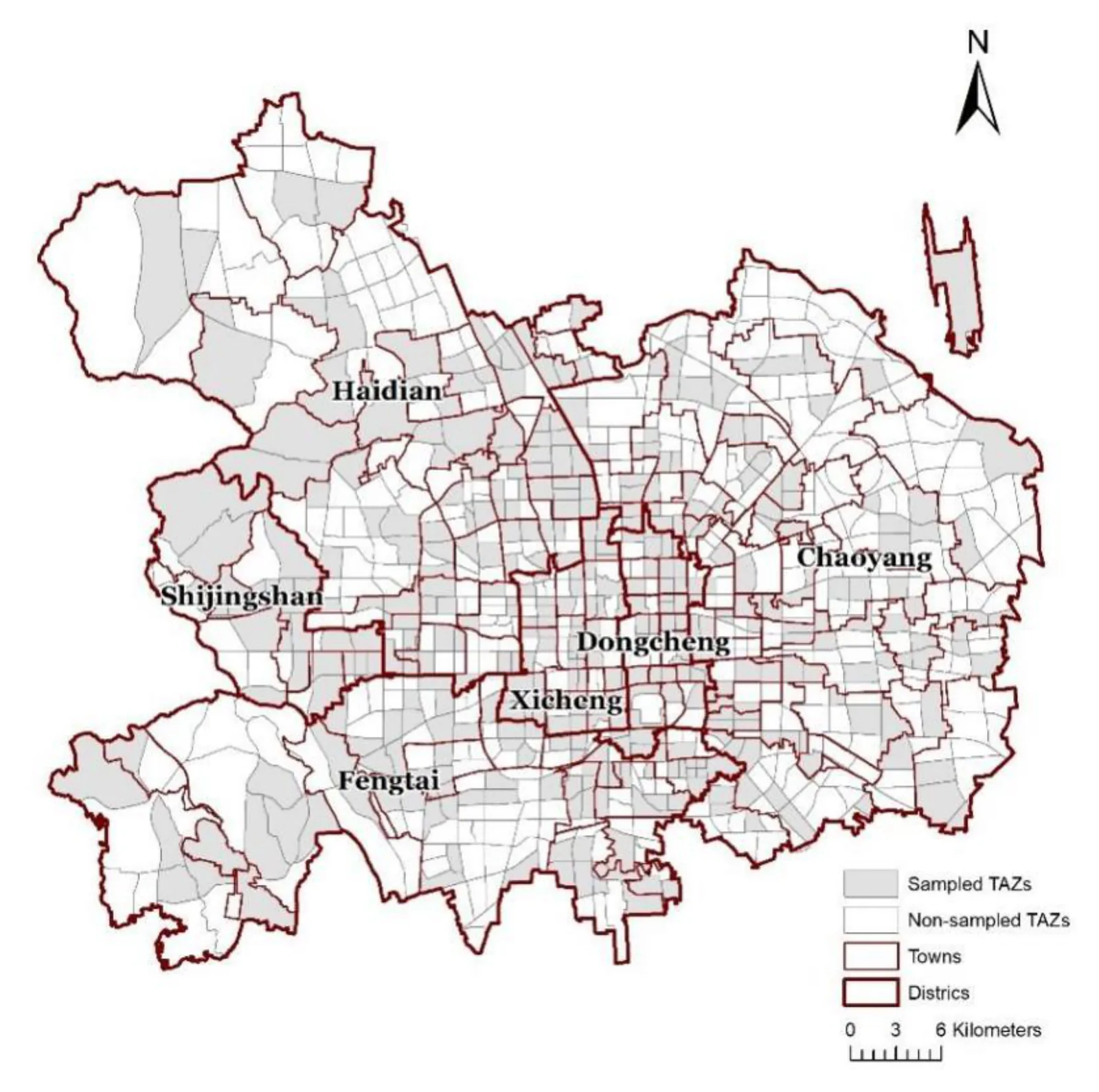
The central urban area of Beijing. Of the 941 TAZs, 523 have no survey coverage (shown in white) and are the target of our prediction.

Our study site is Beijing, the capital of China and one of the country’s megacities, where publicly available fine-scale nSES data remain scarce. Beijing has a highly heterogeneous physical and social environment, and a monocentric urban development pattern has produced uneven development between successive ring roads (Deng & Huang, 2004). The central urban area, which accounts for only about one tenth of the city’s land area but houses roughly half of its population, was selected as the study area (Figure 1). To align with the ground-truth data, we adopt the 2015 TAZ boundaries, matching the year of the Beijing Household Travel Survey used for model training (Section 2.2).

The analysis involves three spatial units, each playing a distinct and non-interchangeable role dictated by data constraints rather than design preference. Neighborhoods (Residential Compounds, Xiaoqu; n = 7,892) are the visual input unit: satellite tiles are cropped to neighborhood boundaries and street view images are collected at neighborhood corner points (Section 2.4). Traffic Analysis Zones (TAZs; n = 941) are the modeling unit, because the ground-truth nSES labels derived from the 2015 survey are released only at this level for privacy reasons; of these, 418 TAZs are covered by the survey and 523 — shown in white in Figure 1 — constitute the prediction target. Townships (Jiedao; n = 130) are used solely as a display unit for visualizing broad spatial patterns (e.g., ring-road gradients), since TAZ-level maps of 941 units can appear visually noisy. Spatially, neighborhoods are nested within TAZs (with boundary-straddling neighborhoods assigned to the dominant TAZ by area), and TAZs are nested within townships, so that aggregation across the three scales is unambiguous.

### 2.2 Survey data

The ground-truth nSES data used for model training and validation are derived from the 2015 Beijing Household Travel Survey, a representative survey originally designed to characterize work–housing location combinations and commuting patterns in central Beijing. The survey covered 418 of the 941 TAZs in the study area, with a sampling rate of 1.36%; the remaining 523 TAZs, shown in white in Figure 1, have no survey coverage and are the target of our prediction. We use this survey as the ground truth for two reasons: it is, to our knowledge, the only publicly accessible dataset that reports nSES-relevant variables at the TAZ level for Beijing, and its 2015 reference year allows strict temporal alignment with the satellite and street view imagery described in Section 2.4 — a point we return to when discussing data timeliness in Section 5.

The survey records both household-level and individual-level socioeconomic attributes. Household-level variables include household size, working population, annual household income, and housing floor area, while individual-level variables include gender, age, hukou type, educational attainment, job type, occupation, and industry. For privacy reasons, respondent locations are released only at the TAZ level, which — together with the resulting need to aggregate all labels to this scale — directly motivates our choice of the TAZ as the modeling unit (see Section 2.1). Section 2.3 describes how these raw survey variables are aggregated into the eight nSES indicators used as training labels.

### 2.3 Calculation of nSES indicators

Having described the survey data source, we now define the eight nSES indicators used as training labels. These indicators are grouped into five categories — household registration (hukou), education, employment, income, and housing — chosen to jointly capture the institutional, human-capital, labor-market, economic, and physical dimensions of neighborhood socioeconomic status. All indicators are computed at the TAZ level because, as noted in Section 2.2, respondent locations are released only at this scale; each neighborhood inherits the indicator values of the TAZ in which it is located, with higher values corresponding to better socioeconomic conditions. Each category is introduced in turn below, starting with hukou, which is specific to the Chinese context and therefore warrants extended explanation for an international readership. Table 2 summarizes the definitions and source variables for all eight indicators.

#### Hukou (household registration)

In mainland China, hukou is the institutional registration system that links a resident to a place of origin and determines local access to public services such as education, pensions, and subsidized healthcare; it has no direct counterpart in most other countries, which is why we describe it in more detail than the remaining indicators. A Beijing hukou therefore confers substantial advantages in access to urban services, and the share of local hukou holders in a neighborhood is widely used as a marker of institutional status in the Chinese urban literature (Song & Smith, 2019). We define the indicator as the percentage of households in a TAZ with at least one member holding a Beijing hukou.

#### Education

Turning from institutional status to human capital, we use two education indicators: the percentage of the population over 25 with tertiary education, and the mean years of schooling. Following the OECD definition, tertiary-educated adults are those aged 25 and above who have completed professional or vocational education following secondary schooling (Organisation for Economic Co-operation and Development, 2018). Mean years of schooling is derived by converting educational attainment levels to years based on the standard academic duration specified by the Chinese Ministry of Education, and serves as a commonly used indicator of aggregate human capital at the neighborhood level.

#### Employment

Moving from human capital to labor-market position, we measure the share of the working population holding high-paying jobs in each TAZ, with higher values corresponding to a lower share of low-paying jobs. Occupation and industry categories follow the Standard of Occupational Classification and the National Economic Classification of Industries issued by China’s National Bureau of Statistics. Following Caban-Martinez et al. (2011), we define high-paying jobs as those in the tertiary sector with occupation types including worker, researcher, office employee, and professional worker, and compute the indicator as the proportion of this group within the total working population of a TAZ.

#### Income

As a direct economic indicator, we use mean annual household income within each TAZ, computed as the sum of household annual incomes divided by the number of sampled households, serving as a direct proxy for the material resources available to residents.

#### Housing

Finally, we characterize the physical dimension of nSES using three housing indicators: mean floor area, mean floor area per capita, and the percentage of commercial housing. Housing has long been recognized as one of the major dimensions of neighborhood deprivation, captured through both internal living space and neighborhood-level housing conditions (Wan & Su, 2016). The first two indicators measure dwelling size at the household and per-capita level, while the third captures housing tenure. Commercial housing (shangpin fang) refers to privately owned, market-rate housing that can be freely traded on the open market, in contrast to affordable or state-allocated housing, which is subject to price controls and eligibility restrictions. A higher share of commercial housing in a TAZ therefore indicates better average housing conditions in the neighborhood.

### 2.4 Satellite and street view images

This section describes the two image data sources used as model inputs — high-resolution satellite imagery and Baidu street view imagery — and explains the rationale for their temporal reference year, which is a key design choice rather than an incidental detail.

#### Satellite imagery

High-resolution satellite imagery for the 7,892 neighborhood units was acquired from Google Earth’s historical archive through a commercial geographic data software (Shuijingzhu), which provides standardized access to Google Earth’s time-series repository. Google Earth was chosen because it offers an accessible archive of historical high-resolution imagery with consistent coverage of Beijing, enabling strict temporal alignment with the 2015 ground-truth survey. We retrieved RGB orthophotos from 2015 with a spatial resolution of 0.11 m, which is essential for capturing micro-scale built-environment features that are not discernible in common open-source multispectral data (e.g., Landsat or Sentinel-2 at 10–30 m resolution). Individual neighborhood images were then obtained by programmatically cropping the global tiles using the precise GIS boundaries of each neighborhood, ensuring full spatial alignment with the survey data.

#### Street view imagery

**Figure 2:**
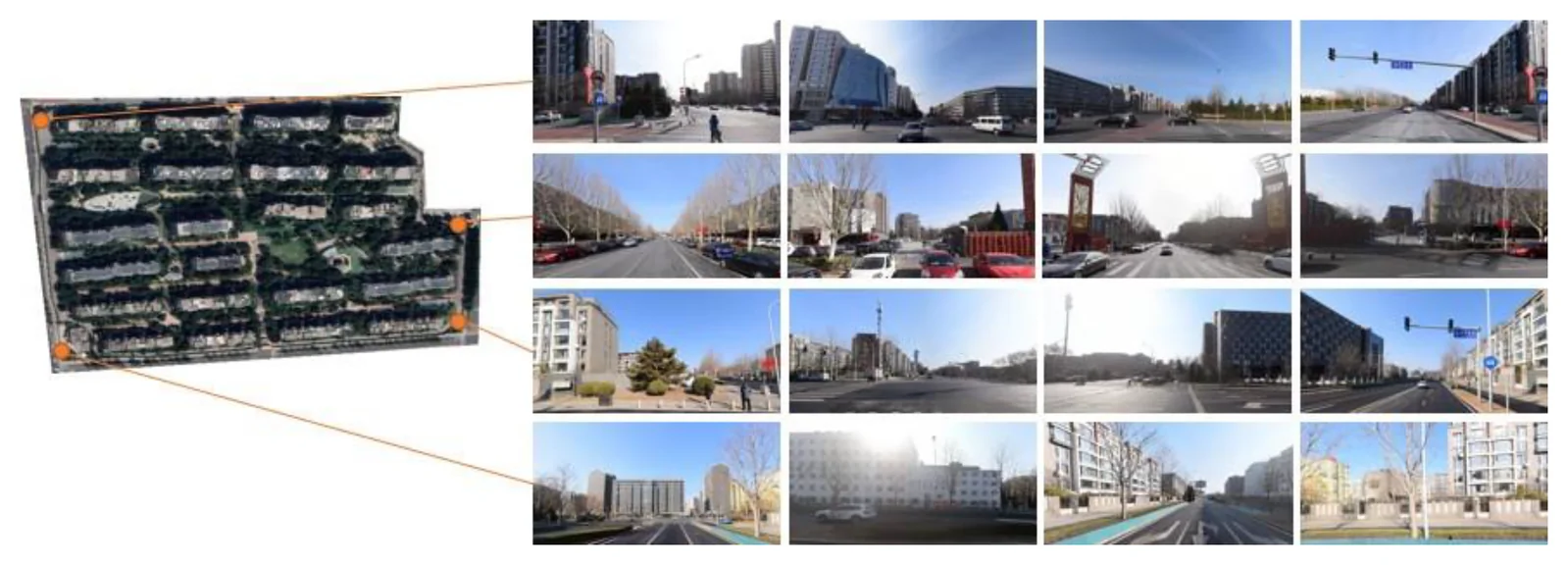
Satellite images of the 7,892 neighborhoods and street view images sampled within each neighborhood, used as model inputs.

Street view images for each neighborhood were obtained from the application programming interface (API) of Baidu Maps, the dominant map service provider in mainland China (see http://api.map.baidu.com/lbsapi/viewstatic.html, accessed 14 January 2023). Baidu was chosen because it offers the most comprehensive street view coverage of Chinese cities, and we used an enterprise-level API key acquired prior to 2018, which allowed bulk download without the strict daily concurrency limits imposed on standard accounts. For each neighborhood, 16 images were obtained at the four corner points of the neighborhood boundary, with four directional views (front, left, back, right) per corner point (see Figure 2); this configuration balances data efficiency against representativeness of the streetscape around the neighborhood (Yue et al., 2022). To align with the 2015 survey reference year, we collected street view imagery from 2015 and 2016, yielding a total of 126,272 images. The full collection process took approximately one month of continuous API access.

#### A note on temporal choice

Our analysis uses imagery from 2015–2016 rather than more recent data, and we emphasize that this is a methodological requirement rather than an oversight. The ground-truth nSES labels come from the 2015 Beijing Household Travel Survey, which is, to our knowledge, the only publicly accessible survey providing nSES-relevant variables at the TAZ level for Beijing; privacy restrictions prevent comparable fine-scale data from being released for more recent years. Pairing current imagery (e.g., 2023) with 2015 labels would introduce a systematic temporal mismatch between predictors and target and bias the regression. We therefore prioritized strict temporal alignment between images and labels, at the cost of using imagery from approximately one decade ago. The broader implications of this choice for the currency of our results are discussed in Section 5.

## 3 Proposed framework

**Figure 3:**
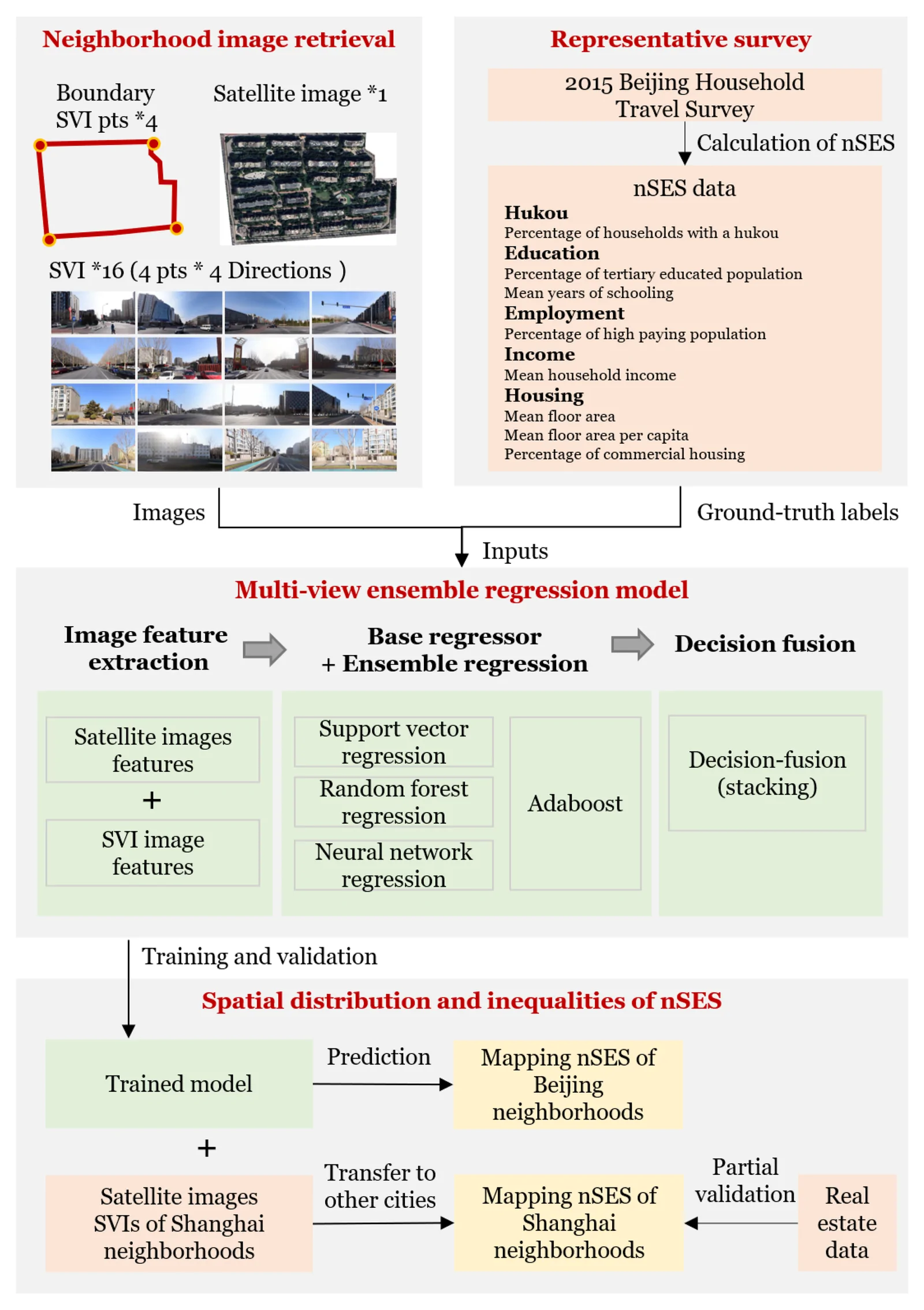
A framework for estimating nSES indicators.

Our framework is designed around three research-driven questions: (i) how to extract informative visual features from imagery given only a small number of labeled TAZs; (ii) how to combine heterogeneous regressors so that no single model is forced to handle all eight nSES indicators; and (iii) how to fuse satellite and street view imagery so that each contributes where it is most informative. Sections 3.1 through 3.4 address these questions in turn. Figure 3 shows the overall architecture of the framework.

### 3.1 Feature extraction

#### 3.1.1 Why extract features rather than train end-to-end?

The 2015 Beijing Household Travel Survey yields ground-truth labels for only 418 TAZs, which is far too small a training set to fit a deep convolutional neural network (CNN) end-to-end without severe overfitting. We therefore adopt a two-stage design that separates the high-capacity visual understanding task — learned once on millions of general-purpose images — from the small-sample regression task learned on our 418 TAZs. A similar transfer-learning strategy — using pretrained deep-learning backbones to extract building-level attributes from street view imagery without end-to-end retraining — has proven effective in related tasks such as estimating building age (Li, Chen, Rajabifard, Khoshelham, & Aleksandrov, 2018). In the first stage, a pretrained visual backbone extracts a compact feature vector from each neighborhood’s imagery; in the second stage (Section 3.2), a lightweight ensemble regressor maps these features to the eight nSES indicators.

For satellite imagery, we use a ResNet-50 backbone pretrained on ImageNet (Russakovsky et al., 2015) to extract a global feature vector from each neighborhood’s cropped satellite tile. Although ImageNet is not remote-sensing data, its low- and mid-level features — edges, textures, and object parts — transfer well to overhead imagery, and fine-tuning the backbone on our small labeled set offered no measurable improvement in preliminary experiments while increasing the risk of overfitting. Each neighborhood is represented by a single satellite feature vector.

For street view imagery, we apply the same ResNet-50 backbone to each of the 16 street view images per neighborhood (four directional views at four corner points; see Section 2.4) and then aggregate the resulting 16 feature vectors into a single neighborhood-level representation by element-wise mean pooling. Mean pooling was preferred over max pooling or attention-based aggregation because the 16 views are sampled at fixed positions rather than chosen for salience, and averaging produces a more stable neighborhood-level representation under the small-sample constraint.

Aggregation to the TAZ level. Because ground-truth labels are only available at the TAZ level (Section 2.1), the neighborhood-level feature vectors are further aggregated to the TAZ to which they belong by taking the mean across all neighborhoods nested within that TAZ. This yields, for each TAZ, one satellite feature vector and one street view feature vector, which are concatenated and passed to the downstream regressor (Section 3.2). Implementation details — backbone layer dimensions, pooling layers, and preprocessing steps — are provided in Appendix A.

#### 3.1.2 Street view pre-processing and visual-dominance features

Street view imagery has intrinsic limitations that a global CNN feature vector alone cannot fully capture. It samples scenes only along drivable roads, and foreground obstructions — particularly roadside trees in summer — can occlude façades and other socioeconomically informative features, introducing systematic bias into downstream predictions. To mitigate these effects, we apply two pre-processing steps before the feature vectors described above are aggregated.

(a) Semantic segmentation and visual-dominance features. Every street view image is passed through a SegFormer-B3 model pretrained on the ADE20K dataset (150 semantic categories), yielding a pixel-level classification of the scene. Pixel-level semantic parsing of street view imagery has become a standard pre-processing step in urban-science applications, including neighborhood physical-disorder assessment (Li, Ma, & Long, 2024) and greenspace analysis (Li, Zhang, Wu, Xue, Wang, Si, & Zhang, 2023); we adopt the same general approach here but tailor the category aggregation to nSES-relevant features. For each image we compute the proportion of pixels belonging to each of eight aggregated categories relevant to the built environment: building, road, vegetation (tree + grass), sky, sidewalk, vehicle, person, and other. These eight visual-dominance ratios — conceptually analogous to the Green View Index and Sky View Factor used in the urban-environment literature — are concatenated with the ResNet-derived global feature vector to form the final per-image representation. This has two advantages: it injects interpretable, semantically meaningful features into an otherwise opaque CNN representation, and it directly supports the interpretability analysis reported in Section 4.4.
(b) Occlusion filtering. Images in which the vegetation class exceeds 60% of all pixels are flagged as severely occluded and excluded from feature aggregation for that neighborhood; if fewer than four usable images remain for a neighborhood after filtering, we substitute the median feature vector of its TAZ to avoid information loss. The 60% threshold was set empirically by visual inspection of a random sample of 500 images and sensitivity-tested between 50% and 70%, with negligible impact on downstream MAPE (< 0.4% variation).
  Incorporating the visual-dominance features reduced the mean MAPE across the eight indicators from 16.8% to 14.5%, and — more importantly for an urban-science audience — produced directly interpretable feature attributions, which we exploit in Section 4.4.

### 3.2 Base regressors and AdaBoost boosting

#### 3.2.1 Why three heterogeneous base regressors?

The eight nSES indicators capture very different aspects of neighborhood life — institutional status, human capital, labor-market position, economic resources, and physical housing — and no single regressor is likely to be optimal across all of them. Rather than searching for a one-size-fits-all model, we adopt three structurally distinct base regressors whose errors are unlikely to be correlated, and later combine them through stacking (Section 3.3). The three regressors are chosen to span complementary inductive biases:

- Support Vector Regression (SVR) with an RBF kernel, a margin-based non-linear learner that is robust to high-dimensional image features and performs well with small training sets (Awad & Khanna, 2015);
- Random Forest Regression (RFR), a bagging-based tree ensemble known for its sensitivity to high-dimensional features, rapid out-of-sample prediction, and minimal hyperparameter tuning (Breiman, 2001; Belgiu & Drăguţ, 2016); RFR has been widely used in image-based regression tasks including surface-temperature estimation (Hutengs & Vohland, 2016), biomass calculation (Mutanga, Adam, & Cho, 2012), and poverty prediction (Zhao et al., 2019);
- a feed-forward Deep Neural Network (DNN) with ReLU-activated hidden layers, a linear output neuron, and dropout regularization to prevent overfitting. The DNN provides a fully learned, non-linear mapping that complements the instance-based geometry of SVR and the piecewise-constant partitioning of RFR.

Heterogeneity across the three families — margin-based, tree-based, and neural — is what allows the downstream stacking layer (Section 3.3) to exploit error diversity rather than merely averaging correlated predictions (Wolpert, 1992).

#### 3.2.2 AdaBoost wrapper

Each of the three base regressors is further wrapped in an AdaBoost procedure (Solomatine & Shrestha, 2004), producing three boosted ensembles — AdaBoost-SVR, AdaBoost-RFR, and AdaBoost-DNN — rather than three bare regressors. Boosting is applied here because it consistently sharpens the per-regressor performance on small, noisy training sets by reweighting “difficult” samples across iterations; prior work has shown that AdaBoost can improve even strong learners such as RF, SVM, and DNN (Jafarzadeh et al., 2021).

At each iteration, samples are resampled according to their current weights, the base regressor is refitted, and the weights are updated according to the regression loss on each sample, with the per-iteration confidence level $\beta_{t}=\bar{L}/(1-\bar{L})$, where $\bar{L}$ is the weighted mean loss. Samples with larger errors receive larger weights in the next iteration, so that the regressor progressively focuses on the harder cases, and the iteration terminates when $\bar{L}\geq 0.5$. The final prediction aggregates the per-iteration predictions using log(1/*β*_*t*_) as weights (details in Appendix A). We adopt linear, mean-square, and exponential loss functions and select the best-performing loss per indicator by inner cross-validation.

### 3.3 Stacking the boosted learners

#### 3.3.1 Why stacking rather than a single boosted learner?

Even after boosting, the three learners remain structurally different and their prediction errors on any given indicator are unlikely to be perfectly correlated. Rather than selecting the single best boosted learner for each indicator — which discards information from the other two — we use stacking (Džeroski & Ženko, 2004) to let the three boosted learners vote through a learned meta-regressor. This is a standard strategy for combining heterogeneous models and is particularly suitable here because the three base families capture genuinely different aspects of the feature space.

#### 3.3.2 Stacking procedure

The training TAZs are split into two disjoint subsets: *X*_part1_ (70%) is used to train the three first-level boosted learners — AdaBoost-SVR (*M*_1_), AdaBoost-RFR (*M*_2_), and AdaBoost-DNN (*M*_3_) — and *X*_part2_ (30%) is used to generate their out-of-sample predictions, which then serve as meta-features for the second-level meta-regressor. This two-part split — rather than in-sample fitting — prevents the meta-regressor from over-trusting predictions that the first-level learners have already seen, a well-known source of leakage in naïve stacking.

Formally, let *F*_1_, *F*_2_, *F*_3_ denote the predictive functions learned by *M*_1_, *M*_2_, *M*_3_ on *X*_part1_. Their predictions on *X*_part2_

$$Y_{k}=F_{k}\left(X_{\text{part2}}\right),\;\;\;k=1,2,3$$

are concatenated into a new three-dimensional feature matrix *X*_new_ = [*Y*_1_, *Y*_2_, *Y*_3_] on which the meta-regressor *F*_meta_ is trained:

$$X_{\text{new}\;}\to F_{\text{meta}\;}\to \;\text{final}\;\text{output}\;$$

We use a Ridge-regularized linear meta-regressor because the meta-feature space has only three dimensions and, at the TAZ scale, a regularized linear combination is both sufficient and robust; more expressive meta-learners offered no measurable improvement in preliminary experiments while increasing the risk of overfitting.

#### 3.3.3 Per-indicator fitting and evaluation protocol

The entire two-level architecture — three AdaBoost-wrapped base learners plus a Ridge meta-regressor — is fitted independently for each of the eight nSES indicators, so that the base-learner weights, AdaBoost iteration counts, and meta-regressor coefficients are free to differ across indicators. This is a substantive modeling choice: as Section 4 will show, indicators differ markedly in their predictability (e.g., hukou is substantially harder than housing floor area), and a shared regressor would be forced into an unhelpful compromise.

All models — the three AdaBoost-wrapped base learners, the stacked ensemble, and the single-view and single-model baselines in Section 4 — are evaluated under an identical five-fold cross-validation protocol on the 418 labeled TAZs, with matched train/test splits. Mean absolute percentage error (MAPE) is reported as the primary metric because it is scale-invariant and allows direct comparison across indicators with different units; R^2^ is reported as a secondary metric. Hyperparameters are tuned via an inner cross-validation loop on the training folds, so that no test-fold information leaks into hyperparameter selection.

### 3.4 Multi-view fusion

#### 3.4.1 Why fuse satellite and street view rather than using one alone?

Satellite and street view imagery carry genuinely different information about a neighborhood. Satellite imagery, viewed from above, captures neighborhood-level layout: building footprints, road patterns, open space, the regularity and density of the built form, and the amount and distribution of greenery. Street view imagery, viewed from ground level, captures pedestrian-scale features that are invisible from above: façade materials and condition, window patterns, the quality of sidewalks and street furniture, and the texture of the streetscape. Our working hypothesis — later verified empirically in Section 4.4 — is that these two perspectives are genuinely complementary rather than redundant, and that different nSES indicators draw on different mixtures of the two. Housing-layout indicators (floor area, commercial housing share) should rely primarily on overhead information, while human-capital indicators (education, high-paying employment) should rely primarily on ground-level streetscape cues. A single-view model cannot exploit this complementarity, which motivates the multi-view fusion design. The principle of combining multi-view imagery through ensemble classifiers has been validated in other urban monitoring tasks, such as crowd congestion estimation from multiple camera perspectives (Li, Sarvi, Khoshelham, & Haghani, 2020); here we extend the idea from classification to regression and from surveillance cameras to satellite and street view imagery.

#### 3.4.2 Fusion strategy

Two standard options exist for combining heterogeneous image features: early fusion, in which satellite and street view feature vectors are concatenated and passed jointly to a single regressor, and late fusion, in which separate regressors are trained on each modality and their predictions are combined at the output stage. We adopt feature-level fusion — a form of early fusion — within the stacking framework introduced in Section 3.2. Specifically, the satellite feature vector and the street view feature vector for each TAZ (together with the visual-dominance ratios from Section 3.1.2) are concatenated into a single joint feature vector, which is then passed to each of the three base learners. The meta-learner operates on the out-of-fold predictions of these three jointly trained base learners, so that the final ensemble prediction for each indicator is a learned combination of base learners that have already seen both modalities.

Why feature-level fusion rather than late fusion? Late fusion would force each single-view model to commit to a prediction before the two modalities can interact, which precludes base learners from discovering cross-modal combinations such as “dense overhead greenery and well-maintained ground-level façades” — a combination that may predict affluent neighborhoods more accurately than either feature alone. Feature-level fusion, by contrast, exposes all cross-modal interactions to the non-linear base learners (Random Forest and DNN) directly, and we found in preliminary experiments that it consistently outperformed a late-fusion variant across all eight indicators (mean MAPE 14.5% vs. 15.7%).

#### 3.4.3 Disentangling modality contributions

A known limitation of feature-level fusion is that, once the two modalities are concatenated, it is no longer obvious how much each contributes to the final prediction — a concern flagged in the urban-science literature when satellite and street view imagery are combined. To address this, we train three variants of the fused model: satellite-only, using only the satellite feature vector; street-view-only, using only the street view feature vector and visual-dominance ratios; and fused, using the concatenation of both. All three variants share identical base learners, meta-learner, and cross-validation protocol (Section 3.2), so that differences in accuracy can be attributed to the modality configuration rather than to modeling choices. The resulting modality-contribution decomposition is reported in Section 4.4 and constitutes part of the interpretability analysis requested for an urban-science audience.

## 4 Results and discussions

This section reports the empirical results in four parts. Section 4.1 evaluates the prediction accuracy of the proposed framework against single-view and single-model baselines. Section 4.2 compares our results against machine-learning methods reported in the prior literature. Section 4.3 visualizes the resulting fine-scale nSES maps for Beijing. Section 4.4 presents the new interpretability analysis — SHAP-based feature attribution, modality-contribution decomposition, and spatial residual mapping.

### 4.1 Accuracy of models

#### 4.1.1 Experimental protocol

As specified in Section 3.3.3, all models are evaluated on the 418 labeled TAZs under an identical five-fold cross-validation protocol, with matched train/test splits across models to ensure a fair comparison. In each fold, 80% of TAZs are used for training and the remaining 20% are held out for testing; the procedure is repeated five times so that every TAZ serves in the test fold exactly once. Mean Absolute Percentage Error (MAPE) is reported as the primary accuracy metric because it is scale-invariant and therefore allows direct comparison across indicators with very different units (percentages, years, square metres, yuan):

$$MAPE=\frac{100\text{\%}}{N}\overset{N}{\underset{n=1}{\sum }}\left|\frac{\hat{y_{n}}-y_{n}}{y_{n}}\right|$$

where N denotes the total number of TAZs, $\hat{y_{n}}$ denotes the predicted value of the TAZ, y_*n*_ denotes the ground-truth value of each TAZ.

#### 4.1.2 Does multi-view fusion improve accuracy?

**Figure 4:**
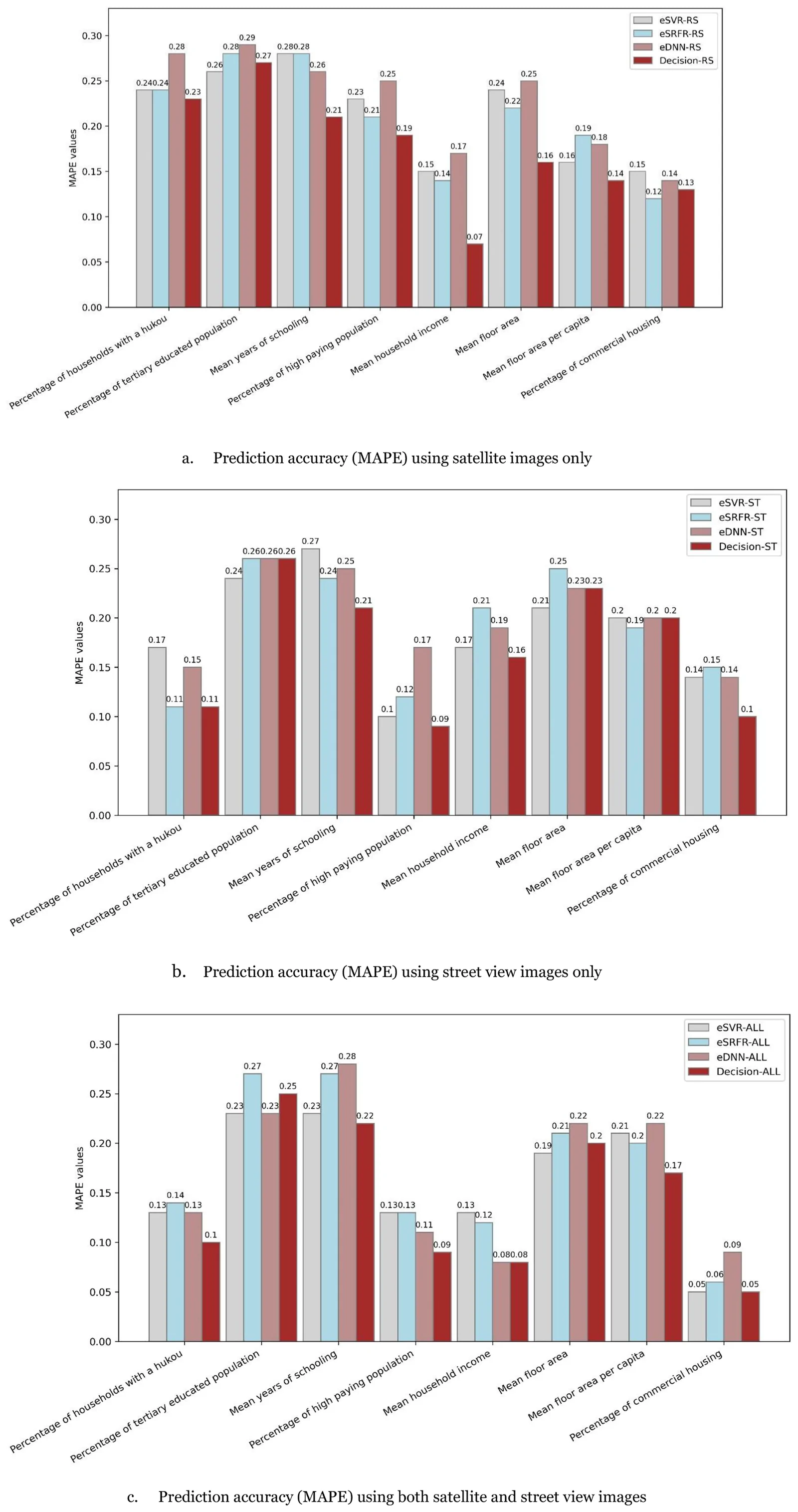
Prediction accuracy (MAPE) across the eight nSES indicators for (a) satellite images only, (b) street view images only, and (c) the multi-view fusion framework proposed in this paper.

The central empirical question of Section 4.1 is whether the proposed multi-view ensemble framework outperforms (i) single-view baselines, which use only satellite or only street view imagery, and (ii) single-model baselines, which use one of the three boosted ensembles alone without stacking. Figure 4 reports the MAPE results across all eight indicators for these comparisons.

##### Effect of data source

Using satellite imagery alone (Figure 4a) yields slightly better accuracy than using street view imagery alone (Figure 4b), with a mean MAPE improvement of 0.5 percentage points (p.p.) across the eight indicators. On average, the multi-view fusion model (Figure 4c) reduces mean MAPE by 3.0 p.p. compared to satellite-only and 2.5 p.p. compared to street-view-only. This average improvement is not uniform across indicators, however. Relative to the satellite-only decision-fusion baseline (DECISION-RS), fusion improves accuracy for percentage of households with a hukou, percentage of tertiary-educated population, percentage of high-paying population, and percentage of commercial housing, but is slightly worse for mean years of schooling, mean household income, mean floor area, and mean floor area per capita (Table 4) — the housing-size and income indicators for which overhead imagery alone already captures most of the signal. Fusion thus improves average accuracy and performs best or near-best for the institutional, human-capital, and housing-tenure indicators, but does not dominate every indicator; we examine the per-indicator modality contributions in Section 4.4.2.

##### Effect of model choice

When multi-view features are used as input (Figure 4c), the stacked decision-fusion model (Decision-ALL) outperforms each of the three single boosted ensembles used in isolation, reducing mean MAPE by 1.8, 3.0, and 2.5 p.p. relative to AdaBoost-SVR-ALL, AdaBoost-RFR-ALL, and AdaBoost-DNN-ALL, respectively. The improvement is consistent across all eight indicators, supporting the argument made in Section 3.3.1 that heterogeneous base learners contribute complementary error patterns that the meta-regressor can exploit.

##### Per-indicator performance

Using the full multi-view fusion model (Figure 4c), four of the eight indicators achieve highly accurate MAPE values at or below the 10% threshold conventionally used to indicate “highly accurate” forecasts (Lewis, 1982): percentage of households with a hukou (10.0%), percentage of high-paying population (9.1%), mean household income (8.0%), and percentage of commercial housing (5.0%). By contrast, when using satellite imagery alone, only mean household income falls below the 10% threshold (7.0%), and when using street view imagery alone, only percentage of high-paying population (9.4%) and percentage of commercial housing (10.9%) achieve comparable accuracy.

These per-indicator patterns hint at the modality-specific contributions that Section 4.4 quantifies in detail: housing-related indicators are easiest from satellite imagery, while labor-market-related indicators are easiest from street view imagery. Indicators related to education and hukou remain the hardest to predict overall, which we revisit in Sections 4.4 and 5.

### 4.2 Comparison of models

Section 4.1 showed that the proposed framework outperforms single-view and single-model baselines within our own experimental setup. A complementary question is how its accuracy compares with that of previously published image-based nSES prediction methods. Because prior studies differ in data sources, target indicators, spatial units, and evaluation metrics, a strict head-to-head comparison is not possible; we therefore report an indicative comparison in Table 3 on the two most commonly reported metrics — R^2^ and MAPE — acknowledging that the underlying datasets and target variables vary across studies.

Across this admittedly heterogeneous set of studies, our framework achieves R^2^ values (85–96%) at the high end of the reported range, and its mean MAPE of 14.5% is competitive with or better than the indicative error rates reported in comparable work (e.g., the 18.3% median relative error in Niu et al., 2020).

Importantly, our framework is the only one in Table 3 that predicts continuous values across eight indicators simultaneously; the other studies predict either a single indicator or a small number of coarse quantile bands. We stress, however, that these numbers should be read as an indicative rather than a definitive comparison, because differences in target variables, spatial scales, and reference populations make cross-study comparisons inherently imperfect.

A more controlled comparison is possible within our own dataset. Table 4 reports per-indicator MAPE for every model variant under the five-fold cross-validation protocol described in Section 4.1: three single-view single-model combinations (SVR, RFR, DNN on satellite imagery only; the same three on street view imagery only); the decision-fusion model applied to each single view (DECISION-RS, DECISION-ST); three single-model variants applied to multi-view features (SVR-ALL, RFR-ALL, DNN-ALL); and the full proposed framework (DECISION-ALL), which applies the decision-fusion meta-regressor to multi-view features.

Three patterns emerge from Table 4, each of which quantitatively substantiates a design claim made in Section 3.

(1) The proposed framework achieves the lowest or joint-lowest MAPE on three of the eight indicators — percentage of households with a hukou (0.10), percentage of high-paying population (0.09, joint with DECISION-ST), and percentage of commercial housing (0.05, joint with SVR-ALL). For the remaining indicators it is competitive but not the single best: percentage of tertiary-educated population is captured slightly better by SVR-ALL (0.23 vs. 0.25), while for mean years of schooling, mean household income, mean floor area, and mean floor area per capita the satellite-only decision-fusion baseline (DECISION-RS) attains the lowest MAPE (0.21, 0.07, 0.16, and 0.14 respectively). This indicates that for the housing-size and income indicators overhead imagery alone already captures most of the signal, consistent with the modality analysis in Section 4.4.2.

(2) Multi-view features improve accuracy for most indicators and base models, though not universally. For example, mean household income is predicted at MAPE 0.15 by SVR-RS (satellite only), 0.17 by SVR-ST (street view only), and 0.13 by SVR-ALL (both), and the decision-fusion meta-regressor reaches 0.08 for DECISION-ALL. The most dramatic improvement is for percentage of commercial housing, where MAPE drops from 0.15 (SVR-RS) and 0.14 (SVR-ST) to 0.05 (DECISION-ALL) — a 66% reduction in error from satellite-only and 64% from street-view-only — confirming that fusing overhead and ground-level perspectives is particularly valuable for housing-tenure classification. The main exceptions are the housing-size indicators such as mean floor area per capita, for which satellite-only features are as accurate as or better than the fused features (Table 4).

(3) The decision-fusion meta-regressor improves accuracy beyond the best individual base model on the same feature set. Within the multi-view group, SVR-ALL, RFR-ALL, and DNN-ALL achieve mean MAPEs of 16.3%, 17.5%, and 17.0% respectively, whereas DECISION-ALL achieves 14.5%. This is consistent with the argument in Section 3.3.1 that heterogeneous base learners contribute complementary error patterns, and that stacking them exploits this diversity rather than merely averaging correlated predictions.

Taken together, the within-dataset comparison in Table 4 complements the cross-study comparison in Table 3: the latter shows that our framework is competitive with the best prior work despite predicting more indicators and continuous values, while the former shows — under matched experimental conditions — exactly where the accuracy gains come from.

### 4.3 Spatial distribution patterns and inequalities in Beijing

**Figure 5:**
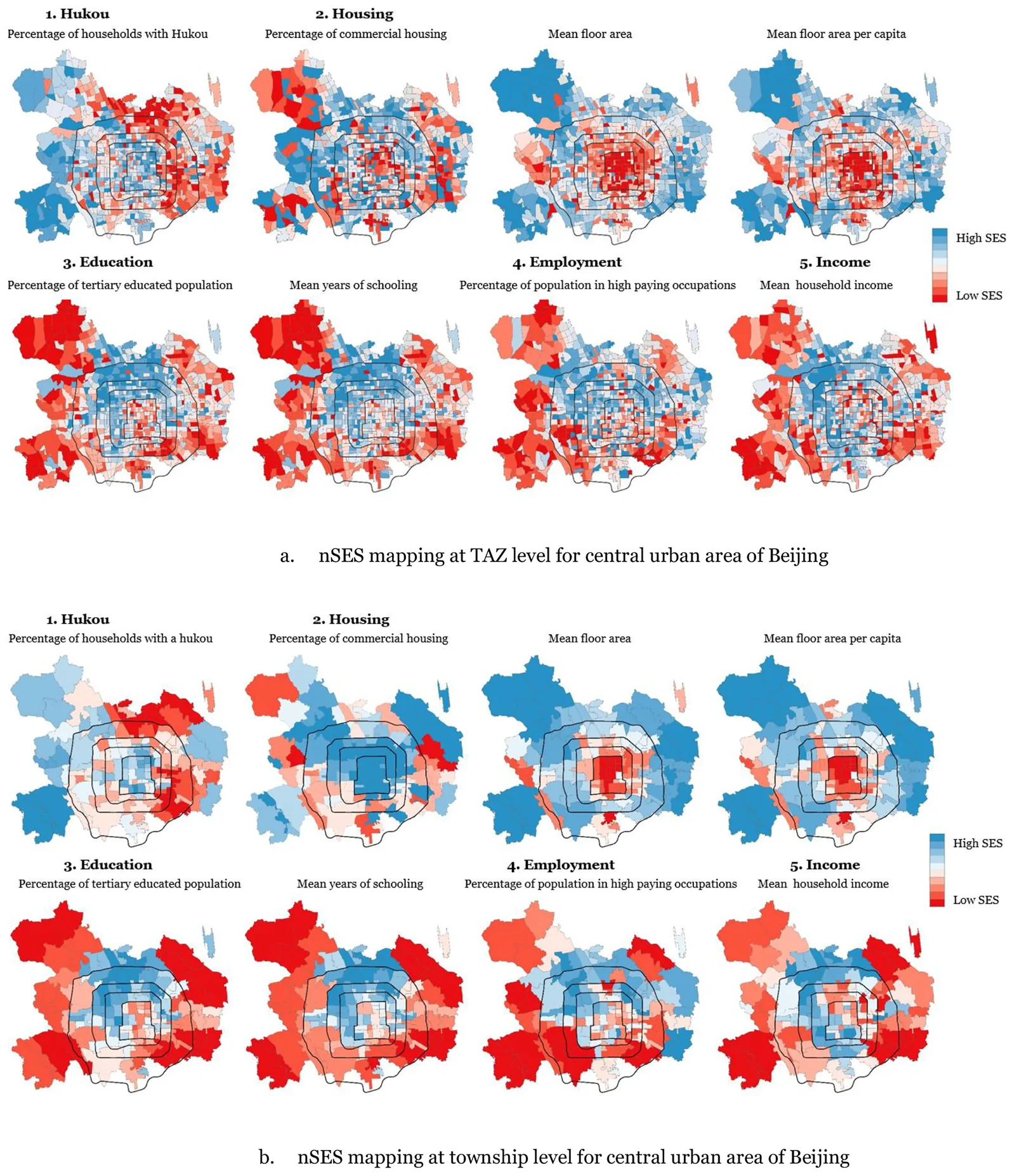
Spatial distribution of the eight nSES indicators in the central urban area of Beijing, predicted by the proposed multi-view ensemble framework: (a) TAZ level (n = 941), and (b) township level (n = 130). The township-level maps are obtained by area-weighted aggregation of the TAZ-level predictions, as specified in Section 2.1.

Having established the accuracy of the proposed framework in Sections 4.1 and 4.2, we now apply it to the full central urban area of Beijing — including the 523 TAZs without survey coverage — to produce fine-scale, full-coverage maps of the eight nSES indicators. This delivers the first empirical contribution of the paper announced in Section 1: a continuous, fine-scale nSES map for a major emerging-economy city where such data are otherwise unavailable. Figure 5 displays the resulting maps at both TAZ and township scales.

nSES mapping at TAZ level for central urban area of Beijing

nSES mapping at township level for central urban area of Beijing

#### 4.3.1 Broad spatial gradients at the township scale

The township-level maps (Figure 5b) reveal coherent, large-scale gradients that align with Beijing’s well-documented monocentric structure and ring-road development pattern. Income is highest in the northern districts and lowest in the eastern, northeastern, southeastern, and western outskirts — a pattern consistent with long-standing accounts of north–south socioeconomic asymmetry in Beijing (Deng & Huang, 2004). This spatial gradient echoes recent fine-scale assessments of urban inequality in Beijing; for instance, Zhang, Wu, Wu, and Li (2021) documented a similarly uneven distribution of community-level greenspace across the central urban area, with greener neighborhoods concentrated in the north and northwest. The eastern fringe shows small pockets of affluence embedded in otherwise lower-income townships, reflecting the ongoing gentrification of formerly industrial districts. Housing-area indicators (mean floor area and mean floor area per capita) show a different gradient: conditions are worst in the historic city center, where land is scarce and dwellings are small, and improve gradually toward the outskirts, irrespective of income. This inverse pattern — high income co-occurring with small per-capita housing area in the inner city — is particularly visible in the old downtown and is consistent with the broader observation that housing quantity and income are decoupled in dense historical cores.

#### 4.3.2 Fine-scale heterogeneity at the TAZ scale

The TAZ-level maps (Figure 5a) reveal a substantially more heterogeneous picture that would be invisible at township scale or in official statistics. Adjacent TAZs often show sharply contrasting nSES profiles: for example, the Xishan No. 1 Courtyard residential complex lies immediately next to a lower-income urban village, and the two are separated by only a few hundred metres yet differ by roughly an order of magnitude on several indicators. Such spatial juxtapositions of well-off and disadvantaged neighborhoods — which the novelist Hao Jingfang (2015) termed “folding Beijing” in her eponymous novella — are consistently recovered by the model and are only visible at the fine spatial resolution our framework is designed to deliver. These fine-grained patterns, imperceptible at the township scale, illustrate the practical value of full-coverage neighborhood-level nSES data for intra-urban inequality research.

#### 4.3.3 Where the visual signal weakens: school-district effects

Not all aspects of nSES are captured equally well by the built environment, and the spatial maps make the limits of image-based inference visible. In Beijing, housing values and the socioeconomic composition of residents are strongly shaped by school district assignments, which are an institutional rather than a physical attribute of a neighborhood. Certain older neighborhoods with unremarkable physical conditions command premium prices — and attract high-income, highly educated residents — solely because they are allocated to top-ranked primary schools. Because our framework relies on the visual appearance of the built environment, it cannot directly observe these invisible institutional attributes, and the model tends to under-predict nSES in such school-district hotspots. We examine this pattern more systematically in Section 4.4 through residual analysis, which identifies school-district neighborhoods as a systematic source of residuals. Despite this limitation, the framework still recovers the dominant spatial gradients across the broader urban fabric, and we return to the implications of this constraint in the Discussion (Section 5).

### 4.4 Interpretability: which visual features drive nSES predictions?

Predictive accuracy alone is insufficient for an urban-science contribution. A practitioner who wishes to use the maps in Figure 5 also needs to know which aspects of the built environment the model is reading, whether satellite and street view imagery genuinely complement each other, and where the visual signal fails. We address these three questions in turn.

#### 4.4.1 Which visual features matter?

To identify the visual features driving each indicator, we apply TreeSHAP (Lundberg & Lee, 2017) to the tree-based component of the stacked ensemble. For each indicator, we rank input features by mean absolute SHAP value and group them into semantic categories using the eight visual-dominance ratios from Section 3.1.2 plus three derived categories (building density, façade heterogeneity, building-layout regularity).

Several clear patterns emerge. Mean household income is driven primarily by building-façade features (mean |SHAP| = 0.38) and building density (0.22), with denser greenery contributing positively — consistent with the well-documented association between intra-urban greening and affluence. This finding aligns with explanatory machine-learning analyses of Beijing’s greenspace, which show that greenspace configuration is a strong correlate of neighborhood-level environmental quality and, by extension, socioeconomic sorting (Li, Zhang, Wu, Xue, Wang, Si, & Zhang, 2023; Zhang, Wu, Wu, & Li, 2021). Percentage of tertiary-educated population is driven by façade heterogeneity (a proxy for architectural style diversity) and sky openness, suggesting that visually varied, open streetscapes are associated with higher educational attainment.

Percentage of commercial housing is dominated by building-layout regularity (mean |SHAP| = 0.41), reflecting the visually standardized appearance of commercial housing complexes compared with older state-allocated or self-built housing. Hukou, by contrast, is the hardest indicator to predict (MAPE = 10.0%) and shows diffuse SHAP attributions with no single feature group exceeding 0.15 — consistent with its conceptual nature as an institutional rather than physical attribute that is only loosely encoded in the built environment.

#### 4.4.2 Are satellite and street view genuinely complementary?

To quantify the contribution of each modality empirically, we use the three model variants introduced in Section 3.4 — satellite-only, street-view-only, and fused — under identical base learners and cross-validation splits. These variants are already reported in Table 4 (rows DECISION-RS, DECISION-ST, and DECISION-ALL respectively), so we can read the modality contributions directly from the accuracy table without requiring an additional figure.

Reading across Table 4, two patterns stand out. First, the two modalities are genuinely complementary rather than substitutable: for most indicators DECISION-ALL matches or outperforms both DECISION-RS and DECISION-ST, and neither modality dominates uniformly; the main exceptions are the housing-size and income indicators (mean years of schooling, mean household income, mean floor area, and mean floor area per capita), for which the satellite-only baseline (DECISION-RS) is as accurate as or slightly more accurate than the fused model. Second, the two modalities specialize across indicators in line with the hypothesis stated in Section 3.4. Satellite imagery contributes more strongly to housing-layout indicators — for example, the MAPE for percentage of commercial housing drops from 0.13 (satellite-only) to 0.05 (fused), a gain of 0.08, larger than the 0.05 gain obtained from the street-only baseline (0.10 → 0.05). Conversely, street view imagery contributes more strongly to human-capital indicators — for percentage of high-paying population, MAPE drops from 0.19 (satellite-only) to 0.09 (fused), a gain of 0.10, while the street-only baseline already achieves the same 0.09 MAPE on its own, indicating that street view alone is sufficient for this indicator and satellite imagery adds no incremental signal. Mean household income is the clearest such case, where satellite imagery alone slightly outperforms the fused model (0.07 vs. 0.08), suggesting that for income prediction the satellite features carry the dominant signal and street-view information adds noise rather than complementary value. Importantly, the complementarity hypothesis was stated a priori in Section 3.4.

#### 4.4.3 Where does the visual signal fail?

A third interpretability question concerns where image-based inference breaks down. We inspected the per-TAZ absolute residuals of the DECISION-ALL model for mean household income (the most interpretable single indicator) and find three categories of systematically high-residual neighborhoods.

The largest residuals occur in school-district hotspots, where inner-city TAZs adjacent to top-ranked primary schools are systematically under-predicted: their high incomes derive from institutional assignment to elite schools rather than from physical attributes the model can see. This quantifies the qualitative observation we made in Section 4.3.3. TAZs dominated by large institutional or non-residential developments university campuses, hospital complexes, and institutional compounds — form a second high-residual category, because the visual features extracted from them reflect institutional rather than residential land use. Boundary TAZs at the urban fringe form a third, smaller cluster, reflecting reduced training signal in peri-urban areas that are under-represented in the labeled survey TAZs. Outside of these three conditions, the residuals are spatially uncorrelated and small, supporting the use of the predicted maps for intra-urban inequality analysis across the bulk of the central urban area. We return to the broader implications of these limitations in Section 5.

Together, the three analyses turn the framework from a black-box regressor into an interpretable instrument: SHAP attribution identifies which features matter, Table 4 shows how the two image sources contribute differently across indicators, and the residual analysis identifies where the framework can and cannot be trusted.

## 5 Discussions

### 5.1 Comparison with other data sources

Our framework relies exclusively on the physical built environment captured by imagery. To further assess the reliability of the image-based predictions, we compare them against an entirely independent data source — Telecom mobile-signaling data — that reflects residents’ digital consumption patterns rather than their physical surroundings. Although socioeconomic status is typically predicted using social-sensing data, prior work has demonstrated strong correlations between the built environment reflected in remote-sensing or street-view imagery and socioeconomic status (Jean et al., 2016; Suel et al., 2019). The question here is whether the nSES patterns we recover from “physical space” align with those implied by “cyberspace behavior”.

We used Telecom mobile-signaling data to infer three TAZ-level indicators for the resident population: average mobile data usage, monthly phone bill, and mobile phone price. Correlation analysis revealed that mobile data usage was strongly correlated with our predicted mean household income, while phone bill and mobile phone price were strongly associated with our predicted percentage of households with a Beijing hukou. Because these two data sources — images and telecom logs — are derived from completely independent pipelines, the consistency between them provides additional evidence that the proposed framework captures genuine socioeconomic signal in the physical built environment rather than incidental visual correlates.

### 5.2 Transferability to other cities

The Beijing results in Section 4 establish the accuracy of the proposed framework within the city where it was trained. A stronger test of its practical value is whether it can be transferred to a different city — one without its own ground-truth survey — and still produce reasonable nSES estimates from imagery alone. This is the core of Contribution (3) announced in the Introduction: the framework is reproducible in data-scarce cities. We select Shanghai as the transfer target because it is China’s most populous city and — crucially — has no publicly available fine-scale nSES survey of the kind we used for Beijing training.

The central urban area of Shanghai includes seven administrative districts and covers approximately 290 km^2^. To obtain neighborhood-level inputs, all neighborhood addresses were first crawled from Anjuke, one of the largest real-estate listing platforms in China: (i) neighborhood names and addresses were submitted to the Amap geocoding API to obtain POI codes; (ii) each POI code was used to query Amap for the corresponding AOI polygon; and (iii) coordinates were converted from GCJ-02 to WGS-84 for spatial alignment with the imagery. Based on the resulting boundaries for 9,801 neighborhoods, satellite images from 2021 were retrieved via Shuijingzhu and cropped to each neighborhood’s AOI boundary. We deliberately used 2021 imagery rather than 2015: this tests whether the framework — trained on historical Beijing data — can analyze current urban conditions in a new city, and demonstrates that the pipeline is not tied to the reference year of the training survey. This is consistent with recent evidence that visual features extracted from time-series street view imagery can track neighborhood-level change across multiple years (Ma, Li, & Long, 2025), suggesting that the learned visual–nSES relationships are not frozen in a single temporal cross-section. Street view images were collected from Baidu Maps under the same sampling criteria used for Beijing, yielding a total of 107,811 images for Shanghai’s neighborhoods. The Beijing-trained DECISION-ALL model was then applied to these Shanghai inputs without any retraining or fine-tuning, so that the transfer result reflects the framework’s ability to generalize rather than its ability to fit a new dataset.

Because no ground-truth nSES data are publicly available for Shanghai, we were unable to verify all eight predicted indicators directly. A limited, same-source proof-of-concept was instead conducted using neighborhood data crawled from Anjuke — including housing type, construction area, and number of households — which can be transformed into two of our predicted indicators: mean floor area and percentage of commercial housing. The Beijing-trained model achieved MAPE values of 0.18 for mean floor area and 0.16 for percentage of commercial housing on the Shanghai data, which are reasonable transfer results considering that (i) the model was trained on a different city, (ii) the imagery reference year differs by six years, and (iii) no local calibration was performed. The Shanghai MAPE for mean floor area (0.18) is comparable to the Beijing in-sample figure (0.20), while the MAPE for percentage of commercial housing (0.16) is notably higher than the Beijing figure (0.05), reflecting the greater difficulty of cross-city transfer for this indicator. Table 5 summarizes the publicly available data required to replicate this transfer procedure for other Chinese cities, including the minimal input data and the two optional validation variables used here.

Consistent with common descriptions of Shanghai’s intra-urban structure, the highest predicted incomes concentrate in central Huangpu, Xuhui, and Jing’an districts, while housing-area indicators follow an inverse gradient — small dwellings in the historic core, larger dwellings in the outer districts — mirroring the pattern observed for Beijing in Figure 5b. The similarity of this cross-city pattern, obtained without retraining, suggests that the visual features the model exploits capture genuinely general aspects of Chinese urban form rather than Beijing-specific artefacts.

## 6 Conclusions

Fine-scale geocoded neighborhood socioeconomic status (nSES) data are foundational inputs for urban studies and spatial inequality research, yet they remain scarce in emerging economies and developing countries that together account for over 70% of the global population. To address this gap, we proposed a multi-view ensemble regression framework that uses visual features from multi-view neighborhood imagery satellite and street view — to estimate eight nSES indicators across five dimensions (hukou, education, employment, income, and housing). Training labels were obtained from the 2015 Beijing Household Travel Survey at the TAZ level, and the full framework was evaluated both within Beijing and through a transfer experiment to Shanghai.

The main findings can be summarized as follows. In terms of data sources, the combined satellite and street-view features outperform single-view features, with an overall average MAPE improvement of 3.0 and 2.5 percentage points compared to satellite and street-view only, respectively. In terms of models, the proposed decision-fusion strategy improves mean MAPE over each of the three single boosted ensembles applied to the same multi-view features (AdaBoost-SVR-ALL, AdaBoost-RFR-ALL, AdaBoost-DNN-ALL) by 1.8, 3.0, and 2.5 percentage points respectively. In terms of indicators, four of the eight achieve highly accurate MAPE values below the 10% threshold: percentage of households with a hukou, percentage of high-paying population, mean household income, and percentage of commercial housing, at 10.0%, 9.1%, 8.0%, and 5.0% respectively. Beyond accuracy, the interpretability analysis in Section 4.4 reveals that satellite and street view imagery play complementary rather than redundant roles: satellite imagery drives the prediction of housing-layout indicators, while street view imagery drives the prediction of human-capital indicators.

Compared with existing image-based nSES methods, this study makes three distinct contributions. First, unlike prior methods that predict only wealth or poverty quantiles (Jean et al., 2016; Suel et al., 2019), our framework recovers continuous numeric values for eight indicators across five dimensions, enabling finer-grained cross-neighborhood comparison. Second, we couple the ensemble with SHAP-based feature attribution and a modality-contribution analysis that explicitly quantify which built-environment features drive each indicator and how the two image sources contribute differently, turning an otherwise black-box regressor into an interpretable instrument for urban analysis. Third, we demonstrate that the Beijing-trained model can be transferred to Shanghai without retraining, addressing the “cold start” problem faced by data-scarce cities and establishing a replicable template for constructing fine-scale nSES datasets elsewhere.

The resulting full-coverage nSES map for the central urban area of Beijing — the first such map publicly available to our knowledge — reveals a highly heterogeneous Beijing, with sharp socioeconomic contrasts between adjacent neighborhoods that would be invisible in coarser administrative statistics. By identifying these fine-scale “micro-pockets” of inequality, the framework provides a foundational data layer for urban planning, residential segregation research, and evidence-based policy targeting.

There are several directions for future work. The framework can be extended to other Chinese cities and to other time periods to enable cross-city and cross-time comparative studies; where the set or quantity of nSES indicators differs, the underlying architecture remains the same and only the input-layer dimensions need to be adjusted. The model is also extensible to features beyond imagery, such as social-media text, POI distributions, or mobility traces, which can be concatenated with the current visual features to provide a more comprehensive picture of nSES. Finally, coupling the fine-scale nSES estimates produced by this framework with health-outcome, education, or environmental datasets would support downstream empirical studies of neighborhood-level disparities. Recent work has already demonstrated that neighborhood infrastructure and built-environment exposures are significantly associated with non-communicable disease risk (Zhang, Liu, Li, Long, Baumgartner, Adamkiewicz, & Bhalla, 2023) and with recurrence risk among cardiovascular patients in Beijing (Liu, Deng, Hu, Chang, Li, Zhang, Su, Liu, & Long, 2023), making the linkage between fine-scale nSES data and health outcomes a concrete and feasible next step — one that, however, falls outside the methodological scope of the present paper.

## Supplementary Material

Appendix A

## Figures and Tables

**Figure 6:**
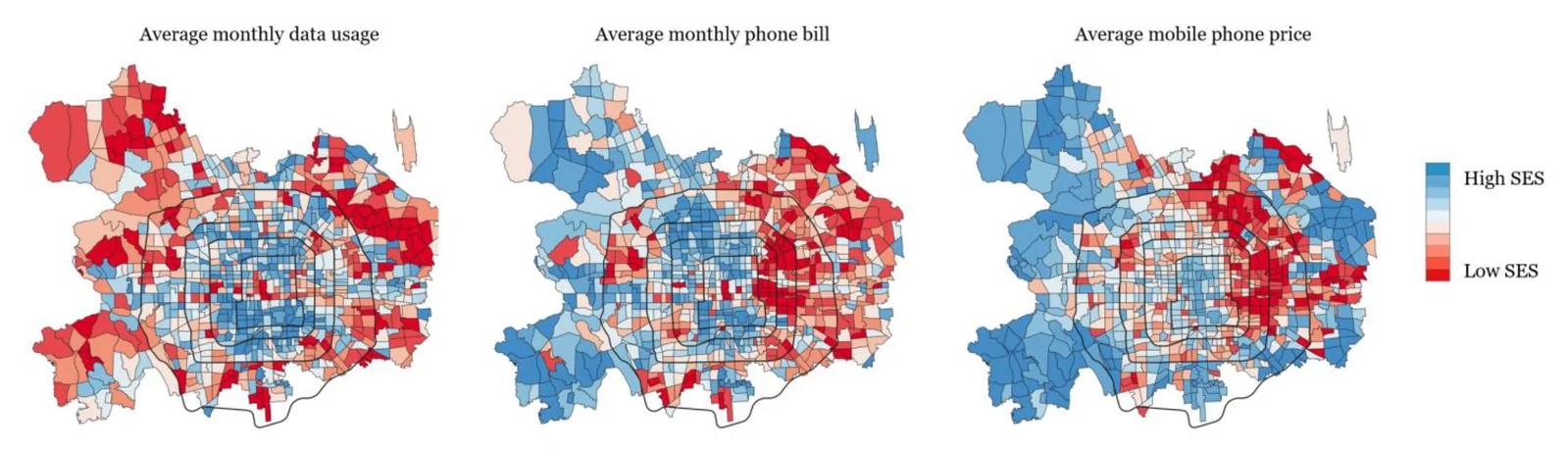
Correlations between image-based nSES predictions and three mobile-signaling variables at the TAZ level.

**Figure 7:**
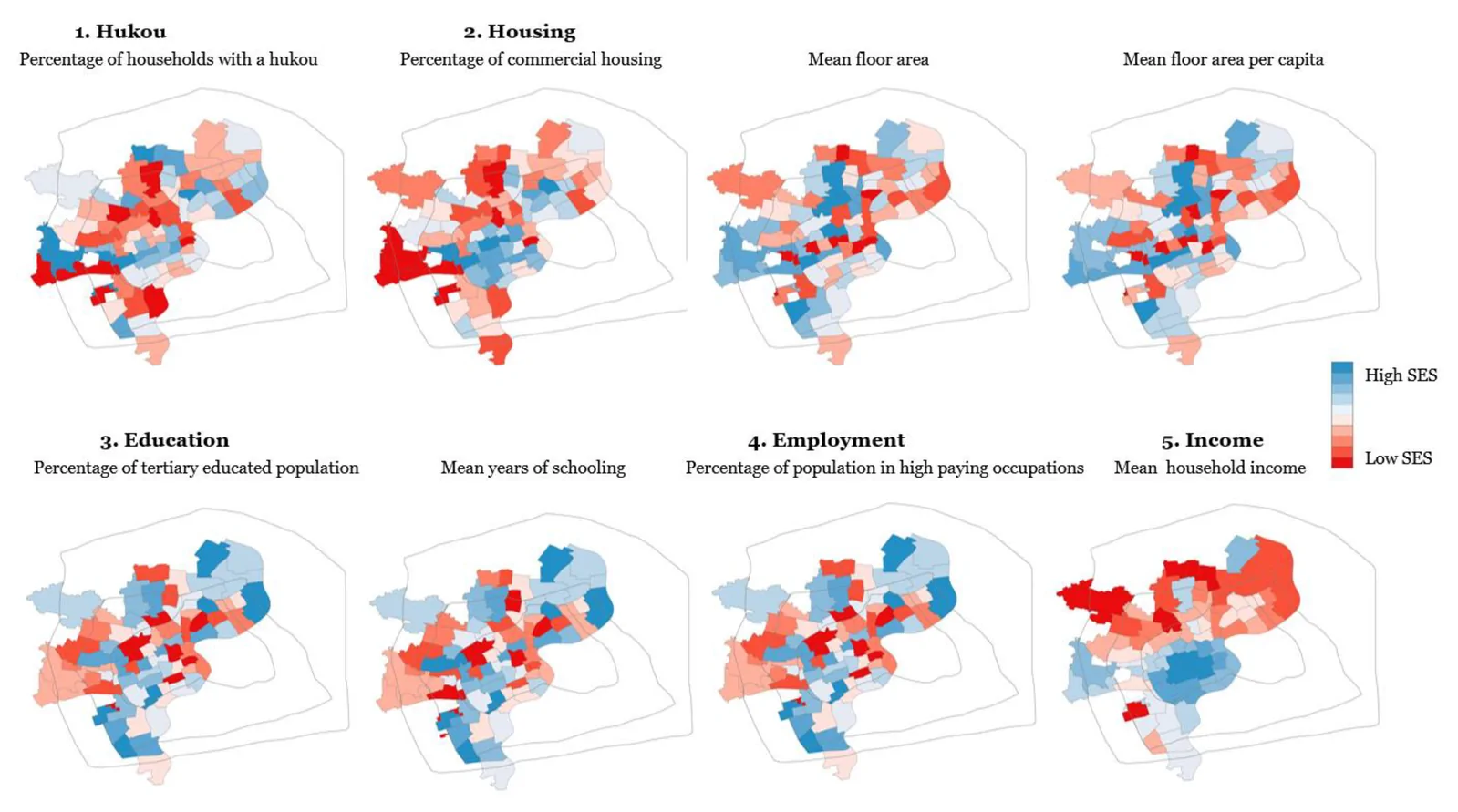
Shanghai nSES indicators at the township level (n = 78), predicted by the Beijing-trained model. TAZ-scale results are not shown because official TAZ boundaries for Shanghai are not publicly available.

**Table 1:** The nSES indicators discussed in the literature.

Data Source	Usage	Relevant Studies
POI data	Assess poverty	Niu et al., 2020
Social sensing data (e.g., mobile signaling data, social media, e-commerce data)	Predict regional population and consumption	Dong, Ratti, & Zheng, 2019; Long & Thill, 2015
Daytime and nighttime satellite images	Reflect intra and inter-urban economic dynamics	Wang, Sutton, & Qi, 2019
Street view images	Inferring demographic makeup of neighborhoods (income, education, race, voting patterns, housing conditions, overall poverty)	Suel, Polak, Bennett, & Ezzati, 2019; Fan, Zhang, Loo, & Ratti, 2023; Gebru et al., 2017; Naik, Kominers, Raskar, Glaeser, & Hidalgo, 2017
Satellite images	Identify areas of poverty, such as slums	Jean et al., 2016; Kuffer, Pfeffer, & Sliuzas, 2016
Combined satellite and street view images	Predict income, overcrowding, and environment deprivation quantiles	Suel, Bhatt, Brauer, Flaxman, & Ezzati, 2021

**Table 2:** Summary of nSES indicators and their definitions.

Category	Indicator	Source variables	Definition
**Hukou**	Percentage of households with a hukou	Hukou type	Percentage of households in a TAZ with at least one member holding a Beijing hukou
**Education**	Percentage of tertiary-educated population	Degree, age	Percentage of TAZ residents aged 25+ with tertiary education (OECD definition)
	Mean years of schooling	Degree, age	Total years of schooling of TAZ residents aged 25+ divided by their population
**Employment**	Percentage of high-paying population	Employment status, occupation, industry, working population	High-paying working population divided by total working population in the TAZ
**Income**	Mean household income	Household income	Sum of annual household incomes divided by the number of households in the TAZ
**Housing**	Mean floor area	Floor area	Sum of floor area of all households divided by the number of households in the TAZ
	Mean floor area per capita	Floor area, household size	Sum of per-capita floor area divided by the number of households in the TAZ
	Percentage of commercial housing	Housing type	Number of commercial housing units divided by the number of households in the TAZ

**Table 3:** Accuracy comparison with other machine-learning methods reported in the image-based nSES literature.

Method	Sources	Model performance
Artificial neural networks	Suel et al. (2019)	R^2^: 66-86%
LASSO regression	Dong et al. (2019)	R^2^: 90-95%
	Fan et al. (2023)	R^2^: 62%
Random forest	Niu et al. (2020)	Median relative error: 18.3 %
Ensemble-based multi-view fusion (this study)	Ours	R^2^: 85-96% MAPE: 14.5%

**Table 4:** Per-indicator MAPE for every model variant, under five-fold cross-validation on the 418 labeled TAZs. The proposed framework (DECISION-ALL) is shown in bold.

Algorithms		Percentage of househ olds with a hukou	Percentage of tertiary educated population	Mean years of schooling	Percentage of high paying population	Mean house hold income	Mean floor area	Mean floor area percapita	Percentage of commerc ial housing
RS images only	SVR-RS	0.24	0.26	0.28	0.23	0.15	0.24	0.16	0.15
	RFR-RS	0.24	0.28	0.28	0.21	0.14	0.22	0.19	0.12
	DNN-RS	0.28	0.29	0.26	0.25	0.17	0.25	0.18	0.14
	DECISION- RS	0.23	0.27	0.21	0.19	0.07	0.16	0.14	0.13
SVIs only	SVR-ST	0.17	0.24	0.27	0.10	0.17	0.21	0.20	0.14
	RFR-ST	0.11	0.26	0.24	0.12	0.21	0.25	0.19	0.15
	DNN-ST	0.15	0.26	0.25	0.17	0.19	0.23	0.20	0.14
	DECISION- ST	0.11	0.26	0.21	0.09	0.16	0.23	0.20	0.10
Image fusion	SVR-ALL	0.13	0.23	0.23	0.13	0.13	0.19	0.21	0.05
	RFR-ALL	0.14	0.27	0.27	0.13	0.12	0.21	0.20	0.06
	DNN-ALL	0.13	0.23	0.28	0.11	0.08	0.22	0.22	0.09
	**DECISION-** **ALL (ours)**	**0.10**	**0.25**	**0.22**	**0.09**	**0.08**	**0.2** **0**	**0.17**	**0.05**

**Table 5:** Data required to transfer the framework to other cities.

Category	Data	Description	Source variable	Data source
**Input data**	Neighborhood boundary	Boundaries were generated by combining neighborhood names and addresses crawled from Anjuke and input to the AMAP geocoding API, AMAP query as well as coordinate conversion to obtain them in batch.	Neighborhood name, address	Anjuke, AMAP API
	Satellite images	Cropping satellite images based on neighborhood boundaries	Neighborhood boundary	Google Earth
	Street view images	16 street view images per neighborhood, captured at the four corner points of the neighborhood boundary with four directional views (front, left, back, right) per corner point	Neighborhood boundary	Baidu street views
**Validation** **data**	Percentage of commercial housing	The total number of commercial households in the statistical unit divided by the total number of households.	Housing type, number of households of each neighborhood	Anjuke
	Mean floor area	First, the mean floor area of each neighborhood is calculated, i.e., the total floor area of the neighborhood divided by the total number of households; then the sum of the average housing area of all neighborhoods in the statistical unit is divided by the total number of neighborhoods.	Total floor area, number of households of each neighborhood	Anjuke
