# Supplementary material for: Measuring Neighborhood Socioeconomic Status from Sky and Street: An Ensemble Learning Framework
Source: Trans Urban Data Sci Technol. Author manuscript; Available in PMC 2026 Oct 20. (PMC7619596; doi:10.1177/27541231261473758)
Supplement: Supplementary file 1 [file EMS217146-supplement-Appendix_A.docx]

**Appendix A: Hyperparameter Settings and Algorithm Details**

This appendix details the specific hyperparameter configurations and implementation libraries for the base regressors and ensemble strategies used in this study. All models were implemented using the Python programming language and the scikit-learn library.

A.1. Support Vector Regression (SVR) The SVR model was implemented using the SVM class from scikit-learn. We utilized a Gaussian Radial Basis Function (RBF) kernel to handle non-linear relationships in the feature space. The optimal hyperparameters were tuned as follows:

- Regularization parameter ($C$): Set to 5. This parameter controls the trade-off between achieving a low error on training data and minimizing the norm of the weights.

- Tolerance ($\epsilon$): Set to 0.05. This defines the margin of tolerance where no penalty is given to errors.

- Kernel coefficient ($\gamma$): Set to 0.01, which defines the influence of a single training example.

A.2. Random Forest Regression (RFR) For the Random Forest model, which fits decision trees on various sub-samples of the dataset, we configured the following parameters to balance performance and computational efficiency:

- Number of trees (ntree): Set to 20. This defines the number of estimators in the forest.

- Features per split (mtry): Set to 1. This represents the number of features to consider when looking for the best split.

A.3. Deep Neural Networks (DNN)

We constructed a standard feed-forward neural network represented as a weighted directed graph. The architecture was configured as a three-layer network to prevent overfitting on the limited dataset:

- Input Layer: Consists of 12 neurons corresponding to the input feature vectors.

- Hidden Layer: Consists of 24 neurons utilizing the ReLU (Rectified Linear Unit) activation function.

- Output Layer: Consists of 1 neuron with a linear activation function for continuous value regression.

- Regularization: A dropout layer was incorporated to improve generalization performance.

A.4. Ensemble and Fusion Strategy

- AdaBoost: For the boosting stage, we set the number of iterations ($T$) to 100 and the error threshold ($\phi$) to 0.05. To further ensure robustness, we applied a pruning strategy where $\nu$ was set to 0.2, meaning only the top 20% of the models were accepted for the final result. At each iteration, sample weights are updated according to the regression loss on each sample; we adopt three candidate loss functions — linear, mean-square, and exponential — and select the best-performing loss for each indicator by inner cross-validation.

- Stacking (Decision Fusion): We employed a stacking strategy where the dataset was split into two parts: 70% ($X_{\text{part1}}$) for training the Level-1 base learners (Adaboost-SVR, Adaboost-RFR, Adaboost-DNN) and 30% ($X_{\text{part2}}$) for training the meta-learner. A Ridge-regularized linear regression (Ridge regression) model was selected as the meta-learner to integrate the predictions from the base learners; because the meta-feature space is only three-dimensional, a regularized linear combination is both sufficient and robust.
